# China CO_2_ emission accounts 2016–2017

**DOI:** 10.1038/s41597-020-0393-y

**Published:** 2020-02-13

**Authors:** Yuli Shan, Qi Huang, Dabo Guan, Klaus Hubacek

**Affiliations:** 1 Energy and Sustainability Research Institute Groningen, University of Groningen, Groningen, 9747 AG Netherlands; 2 Institute of Blue and Green Development, Shandong University, Weihai, 264209 China; 3 Department of Earth System Sciences, Tsinghua University, Beijing, 100080 China; 4 School of International Development, University of East Anglia, Norwich, NR4 7TJ UK; 5 Department of Environmental Studies, Masaryk University, Jostova 10, 602 00 Brno, Czech Republic

**Keywords:** Climate-change mitigation, Environmental impact

## Abstract

Despite China’s emissions having plateaued in 2013, it is still the world’s leading energy consumer and CO_2_ emitter, accounting for approximately 30% of global emissions. Detailed CO_2_ emission inventories by energy and sector have great significance to China’s carbon policies as well as to achieving global climate change mitigation targets. This study constructs the most up-to-date CO_2_ emission inventories for China and its 30 provinces, as well as their energy inventories for the years 2016 and 2017. The newly compiled inventories provide key updates and supplements to our previous emission dataset for 1997–2015. Emissions are calculated based on IPCC (Intergovernmental Panel on Climate Change) administrative territorial scope that covers all anthropogenic emissions generated within an administrative boundary due to energy consumption (i.e. energy-related emissions from 17 fossil fuel types) and industrial production (i.e. process-related emissions from cement production). The inventories are constructed for 47 economic sectors consistent with the national economic accounting system. The data can be used as inputs to climate and integrated assessment models and for analysis of emission patterns of China and its regions.

## Abstract (summary)

**Table:** 

Measurement(s)	carbon dioxide emission • anthropogenic generation of energy
Technology Type(s)	computational modeling technique
Factor Type(s)	year of carbon dioxide emissions
Sample Characteristic - Environment	anthropogenic environment
Sample Characteristic - Location	China

Machine-accessible metadata file describing the reported data: 10.6084/m9.figshare.11793816

## Background & Summary

China’s economic development, energy consumption and associated emissions have entered a “new normal” stage^1^ after a period of rapid development. Economic growth has slowed slightly in recent few years, while more attention has been paid to the optimization and upgrade of economic structures and drivers. Even though China’s emissions have plateaued in 2013 ref. ^2^, it is still the world’s leading energy consumer and CO_2_ emitter, accounting for approximately 30% of global emissions^3^.

After the United States withdrew from the Paris Agreement, China is playing an increasingly important role in global climate change mitigation and emission reduction and has set a series of reduction targets, such as peaking its emissions by 2030 ref. ^4^ and reducing emission intensity by 60%–65% compared with 2005 ref. ^5^. A series of policies, such as those targeting energy structure optimization and renewable energy development, have been implemented and achieved significant results^6^. Renewable and sustainable energy consumption (i.e. primary electricity from hydro power and solar) as well as energy from nuclear power have rapidly increased from 109 million tonnes of standard coal equivalent (tce) (or 3.7% of the total energy consumption) in 2007 to 295 million tce (or 7.1%) in 2017. Meanwhile, the share of coal in total energy consumption has decreased from 75.6% to 65.2% over the same period^7^.

Given these dynamics, up-to-date emission data are a precondition for analysis as well as informed and evidence-based policymaking. The IPCC has proposed a series of emission accounting guidelines for greenhouse gas inventories, including the 1996 version^8^, 2006 version^9^ and 2019 refinement to the 2006 version^10^. In addition, the Paris agreement also requires all parties to provide transparent, accurate, complete, comparable and consistent anthropogenic emission data^11^. However, the Chinese government does not have any official up-to-date inventories. They only estimated overall greenhouse gas emissions for 1994 ref. ^12^, 2005 ref. ^13^, 2010 ref. ^14^, 2012 ref. ^15^, and 2014 ref. ^16^. Some global emission datasets, such as Emissions Database for Global Atmospheric Research (EDGAR), Carbon Dioxide Information Analysis Center (CDIAC), British Petroleum (BP), and the U.S. Energy Information Administration (EIA), cannot also provide sufficiently accurate data estimates for China’s emissions as well. First, these datasets adopt different accounting scopes, methods, data sources, and parameters, leading to incomparable results and discrepancies frequently exceeding 20% ref. ^17^. Second, these global datasets only provide estimates for China’s overall emissions or at most for a few sectors and fuels. They do not provide detailed emission inventories by sectors and fuels for subnational administrative units in China. Third, these datasets do not provide the underlying raw data, making the emission non-transparent and unverifiable. As a result, scholars did lots of repetitive work on emission accounting when analysing China’s emission patterns^18–20^.

Aiming at the above research gap, this study follows a uniform accounting framework to construct the emission inventories of China and its 30 provinces, as well as their energy inventories for the years 2016 and 2017. The inventories are internally consistent and comparable with each other and are compiled based upon the same accounting scope (IPCC administrative territorial scope; energy- plus process-related emissions), methods (sectoral approach and reference approach), data source (official statistical data), and format (17 fossil fuels and 47 economic sectors).

The study provides the most up-to-date emission and energy accounts of China and its 30 provinces. It is a key update of our previous emission dataset 1997–2015 ref. ^17^, as well as an important supplement to official emission estimates. We also publish the activity data, emission factors, and calculation code with the inventories, in order to ensure our data is transparent and verifiable. All data have been uploaded to our open-access dataset: China Emission Accounts and Datasets www.ceads.net for free download.

## Methods

### Accounting scope

Three scopes are widely used in emission accounts^21^. Scope 1 emissions, also called territorial emissions, refer to emissions ‘taking place within national (including administered) territories and offshore areas over which the country has jurisdiction (pageoverview.5)’^8^. In other words, scope 1 accounts for all CO_2_ emissions generated within a country/region boundary, such as energy consumption, production of goods and services and household consumption, as well as emissions from agriculture, forestry, and waste^22,23^. Scope 2 accounts for indirect emissions that relate to electricity/heat consumed within the boundary of a country or region but are produced outside of its boundary. Scope 3 emissions include all other indirect emissions associated with the production of final consumption of a country/region.

Compared with the other two emission scopes, scope 1 emissions describe the physical CO_2_ emissions emitted within a country/region’s boundary and can be used by local governments as a benchmark to design emission reduction policies for their jurisdiction. Therefore, this study follows the IPCC’s administrative territorial scope to account for CO_2_ emissions for China and its 30 provinces.

### Accounting method

We include CO_2_ emissions from both fossil fuel combustion (i.e. energy-related emissions) and cement production (process-related emissions) in the emission accounts. Energy-related CO_2_ emissions are converted from the carbon content in fossil fuels, such as raw coal and gasoline during combustion. We use mass balances to calculate emissions according to the IPCC guidelines^9^, as shown in Eq. 1.

1 $${CE}_{i}={AD}_{i}\times {NCV}_{i}\times C{C}_{i}\times O$$

In the equation, *CE*_*i*_ refers to CO_2_ emissions from fossil fuel *i*. While China’s energy statistical system has 26 types of fossil fuels, we merge them into 17 types due to the small consumption amount and similar quality of certain fuels, shown in Table 1. For example, Naphtha, Lubricants, Paraffin, White spirit, Bitumen asphalt and Petroleum coke are all products of petroleum refineries and account for only 2.8% of total final energy consumption in 2017. *AD*_*i*_ is the “activity data” used for emission estimation. In the case of energy-related emission accounting, *AD*_*i*_ refers to the combustion volume of fossil fuel *i*. *NCV*_*i*_ represents the “net caloric value”, which is the heat value per physical unit from the combustion of fossil fuel *i*. *CC*_*i*_ is the “carbon content” of fuel *i*, which quantifies carbon emissions per net caloric value produced. *O* refers to “oxygenation efficiency”, which represents the oxidation ratio during fossil fuel combustion. The emission factors are collected from our previous study^17,24^ (shown in Table 1), which is estimated based on a wide investigation of 4,243 coal mine samples.

**Table 1:** Fossil fuels and emission factors.

No. (*i*)	Fuels in China’s energy statistics	Fuels in this study	*NVC*_*i*_ (*PJ*/10^4^ *t*, 10^8^ *m*^3^)	*CC*_*i*_ (*t*C/*TJ*)	*CV*
1	Raw coal	Raw coal	0.21	26.32	17.5%
2	Cleaned coal	Cleaned coal	0.26	26.32	10.8%
3	Other washed coal	Other washed coal	0.15	26.32	26.0%
4	Briquettes	Briquettes	0.18	26.32	18.3%
	Gangue				
5	Coke	Coke	0.28	31.38	3.4%
6	Coke oven gas	Coke oven gas	1.61	21.49	15.0%
7	Blast furnace gas	Other gas	0.83	21.49	33.4%
	Converter gas				
	Other gas				
8	Other coking products	Other coking products	0.28	27.45	23.1%
9	Crude Oil	Crude Oil	0.43	20.08	1.1%
10	Gasoline	Gasoline	0.44	18.90	2.0%
11	Kerosene	Kerosene	0.44	19.60	1.2%
12	Diesel oil	Diesel oil	0.43	20.20	1.3%
13	Fuel oil	Fuel oil	0.43	21.10	2.2%
14	Naphtha	Other petroleum products	0.51	17.20	3.9%
	Lubricants				
	Paraffin				
	White spirit				
	Bitumen asphalt				
	Petroleum coke				
	Other petroleum products				
15	Liquefied petroleum gas (LPG)	LPG	0.47	20.00	7.7%
16	Refinery gas	Refinery gas	0.43	20.20	13.5%
17	Natural gas	Natural gas	3.89	15.32	5.5%

Please note that this study only accounts for direct emissions from the consumption of 17 fossil fuels. We do not consider emissions related to electricity/heat consumption to avoid double accounting, as those electricity/heat-related emissions have already been calculated from the production side and allocated to the respective power plants.

Process-related emissions refer to CO_2_ emitted during chemical reactions of industrial production and the CO_2_ emissions are converted from industrial raw materials, rather than fossil fuels. For example, in the production of cement, the calcium carbonate (*CaCO*_3_) is calcined to get calcium oxide (*CaO*). Process-related emissions are converted from the carbon content in *CaCO*_3_, while the emissions from fuel combustion are accounted for in energy-related emissions. In this study, we only include cement production, which accounts for over 70% of China’s process-related emissions^15,25^. According to the IPCC guidelines^9^, process-related emissions can be calculated as follows:

2 $$C{E}_{t}=A{D}_{t}\times E{F}_{t}$$

*CE*_*t*_ refers to process-related emissions from cement production. The activity data, *AD*_*t*_, refers to cement production in the estimation of process-related emissions. *EF*_*t*_ refers to the emission factor, which is 0.2906 tonne of CO_2_ per tonne of cement produced.

### Emissions by sectoral approach and sectoral emission inventories

Energy-related emissions can be calculated by two approaches for a country/region. One is based on sectoral energy consumption data (known as sectoral emissions), the other is calculated based on energy production and supply data, referred to as reference emissions^9^.

Sectoral energy consumption accounts both energy used by final consumers such as agriculture, mining, industry, services and households, and energy used as inputs in energy transformation sectors, in order to produce secondary energy. Most of final energy consumption is from combustion of fossil fuels (excluding a relatively small proportion that is used as raw materials in industrial processes, so called non-energy use), while some of the energy use for energy transformation is not. For example, raw coal is consumed during coal washing in order to obtain cleaned coal, while cleaned coal is transformed to coke during the coking process. There is almost no CO_2_ emitted during such transformations, because the carbon content in raw coal/cleaned coal is transferred to cleaned coal/coke, respectively. Therefore, we exclude such non-combustion inputs in energy transformation when calculating the emissions. Only the fuels combusted in power plants for electricity or heat generation to fuel these processes are included. In this way, in the calculation of sectoral energy-related emissions, we include final energy consumption, energy used for electricity and heat generation, and exclude non-energy use and energy loss.

The current Chinese energy statistical system distinguishes final energy consumption in 47 sectors, which are consistent with the Chinese National Economic System, shown in Table 2 ref. ^26^. We follow this sector definition to construct our emission inventories, which makes it easy to link the inventories to national socioeconomic data. The emissions induced for electricity and heat generation are allocated to the sector “Production and Supply of Electric Power, Steam and Hot Water” and process-related emissions from cement production are allocated to the “Non-metal mineral products” sector as an additional column.

**Table 2:** Economic sectors.

No. (*j*)	Economic sectors
1	Farming, Forestry, Animal Husbandry, Fishery and Water Conservancy
2	Coal Mining and Dressing
3	Petroleum and Natural Gas Extraction
4	Ferrous Metals Mining and Dressing
5	Nonferrous Metals Mining and Dressing
6	Non-metal Minerals Mining and Dressing
7	Other Minerals Mining and Dressing
8	Logging and Transport of Wood and Bamboo
9	Food Processing
10	Food Production
11	Beverage Production
12	Tobacco Processing
13	Textile Industry
14	Garments and Other Fibre Products
15	Leather, Furs, Down and Related Products
16	Timber Processing, Bamboo, Cane, Palm Fibre & Straw Products
17	Furniture Manufacturing
18	Papermaking and Paper Products
19	Printing and Record Medium Reproduction
20	Cultural, Educational and Sports Articles
21	Petroleum Processing and Coking
22	Raw Chemical Materials and Chemical Products
23	Medical and Pharmaceutical Products
24	Chemical Fibre
25	Rubber Products
26	Plastic Products
27	Non-metal Mineral Products
28	Smelting and Pressing of Ferrous Metals
29	Smelting and Pressing of Nonferrous Metals
30	Metal Products
31	Ordinary Machinery
32	Equipment for Special Purposes
33	Transportation Equipment manufacturing
34	Electric Equipment and Machinery
35	Electronic and Telecommunications Equipment
36	Instruments, Meters, Cultural and Office Machinery
37	Other Manufacturing Industry
38	Scrap and waste
39	Production and Supply of Electric Power, Steam and Hot Water
40	Production and Supply of Gas
41	Production and Supply of Tap Water
42	Construction
43	Transportation, Storage, Post and Telecommunication Services
44	Wholesale, Retail Trade and Catering Services
45	Other Service Sectors
46	Urban Residential Energy Usage
47	Rural Residential Energy Usage

In this way, we construct the sectoral emission inventories of China and its 30 provinces by 47 economic sectors (rows), 17 fossil fuels (columns), and the cement production process (the 18^th^ column).

### Emissions by reference approach and reference emission inventories

According to the IPCC guidance, the reference method “*is a top-down approach, using a country’s energy supply data to calculate the emissions of CO*_2_ *from combustion of primary fossil fuels. The reference approach is a straightforward method that can be applied on the basis of relatively easily available energy supply statistics (Volume* 2*, Chapter 6, Page 5)*”^9^. The reference emissions are an important supplement to sectoral emissions and can be used for verification. The reference emissions can be estimated with reference energy consumption ($A{D}_{ref-i}$), which is shown in Eq. 3. We exclude energy loss and non-energy use in the calculation of reference energy consumptions and emissions due to the same reason as in the sectoral approach.

3 $$\begin{array}{lll}A{D}_{ref-i} & = & Indigeous\,production+imports-exports\,+(Moving\,in\,from\,other\,provinces\\  &  & -Sending\,out\,to\,other\,provinces)\pm stock\,changes-\,Non\,energy\,use-Loss\end{array}$$

We only consider three primary fossil fuels - raw coal, crude oil, and natural gas - when calculating reference emissions and energy consumption. The basic assumption is that all secondary fuels are transformed from primary fuels, with their carbon content coming from the primary fuels. If we consider the country/region as a black box, the reference consumption of three primary fuels contains the overall carbon content consumed within the black box, no matter how they are transformed or circulated among energy types or sectors.

The reference emission inventories are constructed for the three primary energy types (raw coal, crude oil, and natural gas) and for six sub-items at the country level (indigenous production, import, export, stock change, loss, and non-energy use), eight sub-items at the provincial level (plus moving in from and out to other provinces).

### Activity data

Energy activity data are collected from China and its provinces’ Energy Balance Tables, which are published in the China Energy Statistical Yearbook 2017 and 2018. The Energy Balance Table presents comprehensive energy flows and utilization including production, transformation, final consumption, loss and others.

Final energy consumption in the Energy Balance Table includes only eight sectors: “Farming, Forestry, Animal Husbandry, Fishery and Water Conservancy”, “Industry”, “Construction”, “Transportation, Storage, Post and Telecommunication Services”, “Wholesale, Retail Trade and Catering Services”, “Other Service Sectors”, “Urban Resident Energy Usage”, and “Rural Resident Energy Usage”. We then expand the “Industry” sector to 40 sub-sectors ($j\in [2,41]$ in Table 2) according to the “Industry Sectoral Energy Consumption Table (ISECT)”. The country level ISECTs are collected from the China Energy Statistical Yearbook 2017 and 2018, while the provincial ISECTs are collected from each provinces’ statistical yearbooks. Due to a lack of data, the nine provinces of Hebei, Shanghai, Jiangsu, Zhejiang, Shandong, Guangxi, Hainan, Sichuan, and Guizhou do not have ISECTs. We use data from previous years instead, assuming their economic structure remained unchanged.

The cement productions of China for the years 2016 and 2017 are collected from the China Statistical Yearbook 2017 and 2018, respectively. Provincial production is collected from each province’s statistical yearbook 2017 and 2018.

The description of the methods on constructing the emission inventories are an expanded version of our previous work^17^.

## Data Records

The dataset “China CO_2_ emission accounts 2016–2017” is made public under Figshare^27^. A total of 1,172 data records (emission and energy inventories) are contained in the dataset. Of these,

1. two are national energy inventory (2016 and 2017) [File “China national energy inventory, 2016–2017”];
2. 60 are provincial energy inventory (30 provinces, 2016 and 2017) [File “China provincial energy inventory, 2016–2017”];
3. two are national sectoral emission inventories (2016 and 2017) [File “China national CO_2_ emission inventory (sectoral approach), 2016–2017”];
4. two are national reference emission inventories (2016 and 2017) [File “China national CO_2_ emission inventory (reference approach), 2016–2017”];
5. 60 are provincial sectoral emission inventory (30 provinces, 2016 and 2017) [File “China provincial CO_2_ emission inventory (sectoral approach), 2016–2017”];
6. 60 are provincial reference emission inventory (30 provinces, 2016 and 2017) [File “China provincial CO_2_ emission inventory (reference approach), 2016–2017”].

**Fig. 1:**
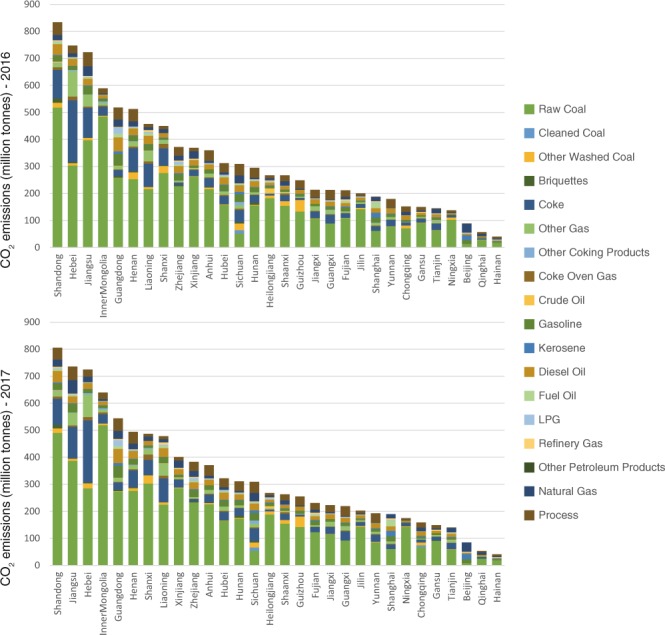
Provincial CO_2_ emissions by fossil fuels and cement production, 2016–2017.

The emission and energy inventories provide detailed CO_2_ emitted from 17 types of fossil fuel used in 47 economic sectors for China and its 30 provinces. Further analysis of emission patterns can be conducted based on the data. For example, Fig. 1 shows the 30 provinces’ CO_2_ emissions by fuels and cement production in 2016 and 2017, which is drawn with Data Citation 1, File “China provincial CO_2_ emission inventory (sectoral approach), 2016–2017”.

From Fig. 1, we find that Shandong was the top emitting province followed by Hebei and Jiangsu in 2016. Jiangsu surpassed Hebei and became the second largest emission source in 2017. The top emitting provinces are either rich regions (such as Jiangsu), or have many manufacturing or energy producing industries (such as Hebei and Inner Mongolia), or large populations (such as Henan). When considering the emission intensities (per GDP emissions), Ningxia (0.43 tons per thousand Yuan), Xinjiang (0.38) and Shanxi (0.34) are the top three provinces in 2016, indicating carbon-intensive economic structures of these provinces. The top three provinces having high emission intensities in 2017 are Ningxia (0.51), Inner Mongolia (0.40), and Xinjiang (0.37). In contrast, Beijing (0.035 in 2016 and 0.030 in 2017), Guangdong (0.064 in 2016 and 0.060 in 2017), and Shanghai (0.067 in 2016 and 0.062 in 2017) have the smallest emission intensities among all the provinces in 2016 and 2017. These three provinces are the most developed regions in China and their economic structure is dominated by service sectors. When considering the structure of emission sources, coal and its related products are still dominating in most provinces’ energy structure, especially in Ningxia (95.3%), Inner Mongolia (94.5%), Hebei (91.3%), and Shanxi (91.2%) in 2017. This is mainly determined by the natural endowment and industrial structures. In contrast, Beijing (40.0%), Qinghai (20.4%), and Tianjin (12.2%) have a relatively higher share of natural gas-related emissions than other provinces.

## Technical Validation

### Uncertainties

The uncertainty of emission accounts may come from various sources. According to the IPCC^9^, activity data, emission factors, lack of completeness, lack of data, and measurement errors may lead to different levels of uncertainties. However, due to technical issues, some of the uncertainties are not quantifiable. For example, the measurement error “is random or systematic, resulting from errors in measuring, recording and transmitting information; inexact values of constants and other parameters obtained from external sources (Volume 1, Chapter 3, Page 11)”^9^, and such uncertainty exists in every step when developing emission accounts. In this study, we discuss the uncertainties from activity data and emission factors, which are the major quantifiable uncertainty sources in emission accounts. The uncertainty analysis acknowledges the limitations and potential inaccuracy of our emission inventories and provides a more accurate illustration of our emission estimates.

Activity data is one of the most important sources of uncertainty of emissions. Due to the dual system and poor quality of the energy statistics^28^, China’s energy consumption data may have a Coefficient of Variation (CV, the standard deviation divided by the mean) ranging from 5% to 30% ref. ^29^ depending on sectors: e.g., for electricity generation the CV is 5% ref. ^30,31^; industry and construction 10% ref. ^9,32^; transportation 16% ref. ^33^; residential 20% ref. ^9^; and primary industry 30% ref. ^34^. Despite China having modified its national energy consumption data three times since 2000, there is still a 5% difference between the national and aggregated provincial energy data^35^.

The uncertainties in China’s emission factors have been widely discussed in the research community^36–39^. Despite default emission factors of China being published based on IPCC guidelines, the government and some research groups published their own factors, such as: National Bureau of Statistics (NBS), NDRC, Initial National Communication on Climate Change (NC1994), Second National Communication on Climate Change (NC2005), Multi-resolution emission inventory for China (MEIC), UN-China, and UN-average. Our previous comparisons of eight different factor sources found that the coefficient of variation of fuels’ emission factors ranged from 1.1% (crude oil) to 33.4% (other gas). Liu *et al*.’s^24^ emission factors used in this study is relatively low compared to the other sources, but higher than MEIC and NC1994 values. The coefficients of variation for different fossil fuels are presented in Table 1.

We applied Monte Carlo simulation, which is a technique recommended by the IPCC^9^ to propagate the uncertainties from activity data and emission factors and calculate the integrated uncertainty of the entire emission inventory. The technique first assumes distributions (probability density function) for both activity data and emission factors. In this study, we assume that both variables follow a normal distribution^24^ and their standard deviations are discussed above. Then, the technique generates a mass of random samples (100,000 times in this study) of the two variables, meaning that 100,000 independent estimations of emission can be calculated. The 97.5% uncertainty range is calculated as the 97.5% confidence intervals of the 100,000 estimations. Compared with energy-related emissions, process-related emissions have relatively lower uncertainties due to less parameters and simpler calculation methods^24^. We only quantify the uncertainties from energy-related emissions in this study.

**Fig. 2:**
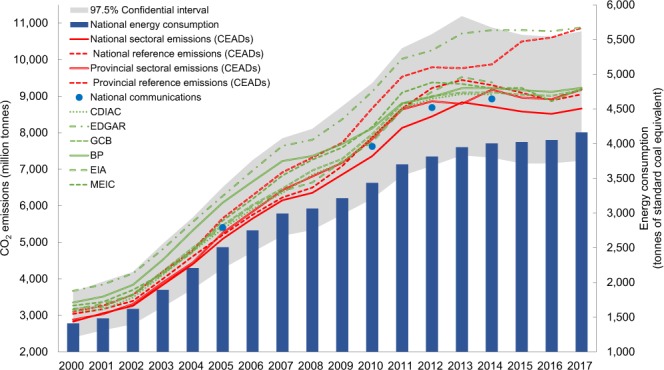
Uncertainties and comparisons with other datasets.

The results show that the uncertainty range for energy-related emissions falls within (−15.1%, 30.8%) in 2016 and (−15.0%, 30.3%) in 2017 at a 97.5% confidential level. The 97.5% confidential interval of our estimates are shown in Fig. 2 (the grey area). We simulate the uncertainty range of a variable by keeping the others constant. The results show that the uncertainties from the activity data are (1.9%, 12.8%) in 2016 and (1.9%, 12.7%) in 2017, while those from the emission factors are higher: (−14.5%, 29.6%) in 2016 and (−14.6%, 29.6%) in 2017.

### Comparisons with existing emission datasets

In order to verify our emission accounts, we compared our estimates with other emission datasets, as shown in Fig. 2. While we found that our estimates produce relatively lower emissions, our estimates are very close to China’s official emissions, with gaps ranging between −5.81% to 1.98%. Our national sectoral emissions were 5.81%, 3.49%, 2.79%, and 2.36% lower than the official emissions of 2005, 2010, 2012, and 2014, respectively. Meanwhile, our provincial aggregated emissions were 2.73% lower for 2005, and 2.57%, 1.98%, and 2.85% higher than the official national emissions for 2010, 2012 and 2014, respectively.

As these existing emission datasets only provide the total emissions of China, we cannot make a further comparison of the estimates at the provincial or sector level. That is to say that our estimates provide the most up to date and comprehensive emission inventories of China and its provinces, and is an important supplement to the existing emission estimates as well as the official emission inventories.

### Limitations and future work

Our datasets have several limitations, but we will work on these limitations in the future to improve the accuracy of China’s emission accounts.

1. We only include the emissions from fossil fuel combustion and cement production. There are many other components in scope 1 direct emissions, such as emissions from waste treatment and landfills and other industrial processes. Despite these components only accounting for a small proportion of overall emissions (less than 4% in 2012 ref. ^15^), missing them makes our inventories incomplete. In the future, we will expand our accounting scope to achieve more comprehensive emission inventories for China and its regions.
2. We adopt the national average emission factors to calculate each province’s fossil fuel-related emissions. The provinces may have heterogeneity in energy quality and utilization efficiency, and using the national average factors for all provinces may lead to additional uncertainties at the provincial level. Similar uncertainties also exist in the process-related emission accounts. The emission factor for cement production is estimated based on the national average clinker-cement ratio and clinker emission factors^24^. Due to the variance in production technologies in different regions, the clinker emission factors and clinker-cement ratios would be different as well^25^. Our future work will investigate regional specific emission factors for China to achieve more accurate emission data for China’s provinces.
3. Due to data accessibility, nine provinces do not have sub-sectoral energy consumption of their industry. The current version uses historical data, which is collected from the national economic census of 2008 ref. ^40^. We thus assume that the economic structure of these provinces remained the same over the past 10 years. In the future, we will investigate these provinces to obtain more updated sectoral energy data for them.

## Data Availability

The MATLAB Code used to generate the emission inventories with energy inventories is published below for transparency and verifiability. We take Anhui 2017 as an example. % Copyright 2019 Yuli Shan. All rights reserved. *% Read emission factors and energy data from the excel files* NCV = xlsread(‘Emission factors’, ‘NCV’, ‘A2:Q2’); *%NCV*_*i*_ *refers to* Table 1 *in Shan, et al*.^17^ NCV = repmat(NCV,68,1); CC = xlsread(‘Emission factors’, ‘CC’, ‘A2:Q2’); *%CC*_*i*_ *refers to* Table 1 *in Shan, et al*.^17^ CC = repmat(CC,47,1); O = xlsread(‘Emission factors’, ‘Oxygenation Efficiency’, ‘B2:R48’); *%O*_*ij*_ *refers to* Table 3 *in Shan, et al*.^17^ Energy = xlsread(‘Province Energy inventory 2017’, ‘Anhui’, ‘B3:R70’); *% Convert physical energy consumption to calories* E_PJ1 = Energy.* NCV; E_PJ1(1,:) = sum(E_PJ1(2:48,:)); E_PJ2 = E_PJ1; *% Remove non-energy use from the total consumption* E_NE = zeros (68,17); E_NE (23:27, 1:17) = repmat (E_PJ1(65,:),5,1) .* E_PJ2(23:27,:) ./ repmat (sum(E_PJ1(23:27,:)),5,1); E_NE (:,16) = E_PJ2 (:,16); E_NE(isnan(E_NE)) = 0; *% Include energy combustion consumption during transformation process* E_Trans = zeros (68,17); E_Trans (40,:) = E_PJ1(52,:) + E_PJ1(53,:); E_Trans (44,10:13) = E_PJ1(67,10:13) + E_PJ1(68,10:13); E = E_PJ2(1:48,:)-E_NE(1:48,:) + E_Trans(1:48,:); E(1,:) = sum(E(2:48,:)); *% Calculate the energy-related emissions* CO2_E = E (2:48,:) .* CC .* O/100; CO2_E(CO2_E < 0) = 0; *% Calculate the process-related emissions* EF_cement = 0.2906; Prod_cement = 133.94; *%Prod_cement here refers to Anhui’s cement production in* 2*017* CO2_cement = Prod_cement .* EF_cement; *% Construct the emission inventory* CO2 = zeros (48,19); CO2 (2:48,1:17) = CO2_E; CO2 (28,18) = CO2_cement; CO2 (:, 19) = sum(CO2,2); CO2 (1,:) = sum(CO2(2:48,:))
